# First Application of Fecal Microbiota Transplantation in Adult Asperger Syndrome With Digestive Symptoms—A Case Report

**DOI:** 10.3389/fpsyt.2022.695481

**Published:** 2022-03-15

**Authors:** Hong-Li Huang, Hao-Ming Xu, Yan-Di Liu, Di-Wen Shou, Hui-Ting Chen, Yu-Qiang Nie, Yong-Qiang Li, Yong-Jian Zhou

**Affiliations:** Department of Gastroenterology and Hepatology, Guangzhou Digestive Disease Center, Guangzhou First People's Hospital, School of Medicine, South China University of Technology, Guangzhou, China

**Keywords:** fecal microbiota transplantation (FMT), Asperger syndrome (AS), diarrhea predominant irritable bowel syndrome, metagenomic sequencing, untargeted metabolomics analysis

## Abstract

Asperger syndrome (AS) is a chronic neurodevelopmental disorder. Although all of the clinically diagnosed cases display normal intelligence and speech functions, barriers in social interaction and communication seriously affect mental health and psychological function. In addition to traditional psychological/behavioral training and symptomatic medication, in-depth studies of intestinal microbiota and mental health have indicated that probiotics (e.g., *Lactobacillus rhamnosus*) can effectively reduce the occurrence of AS. Fecal microbiota transplantation (FMT) is a type of biological therapy that involves the transplant of intestinal microbiota from healthy donors into the patient's gastrointestinal tract to improve the gut microenvironment. In this case report, we describe the first case of adult AS treated with FMT. The patient suffered from diarrhea-predominant irritable bowel syndrome for 6 years with symptoms of diarrhea and abdominal pain. After three rounds of FMT, the diarrhea and abdominal pain were significantly improved. Moreover, the symptoms of AS were also significantly ameliorated. We found that FMT changed the structure of the intestinal microbiota as well as the patient's serum metabolites, and these changes were consistent with the patient's symptoms. The metabolites may affect signaling pathways, as revealed by Kyoto Encyclopedia of Genes and Genomes enrichment analysis. The changes in microbial metabolites following FMT may affect other regions (e.g., the nervous system) via the circulatory system, such that the bacteria-gut-blood-brain axis may be the means through which FMT mitigates AS.

## Introduction

First described by Hans Asperger in 1944, Asperger syndrome (AS) is a neurodevelopmental disorder that begins in childhood. While AS patients have normal vocabulary and cognitive functions, they experience social difficulties and stereotyped and repetitive behaviors. To a certain extent, AS is considered a subtype of autism spectrum disorder (ASD) (1). Gut microbiota-targeted therapy has been shown to affect psychiatric disorders (2). Fecal microbiota transplantation (FMT) is an emerging therapy that involves transplanting fecal microbiota isolated from healthy donors into the patient's gastrointestinal tract to reconstruct their gut microbiota and restore microbial homeostasis (3). FMT has been reported to be an effective treatment for recurrent *Clostridium difficile* infection. Notably, gut microbiota play an important role in the pathophysiology of neurological and psychiatric disorders via the microbiota-gut-brain axis. FMT targets gut microbiome dysbiosis and improves neurological and psychological symptoms (4). Moreover, the early administration of probiotics (*Lactobacillus rhamnosus*) can reduce the risk of developing AS (5). Given that the successful alleviation of ASD with FMT has been observed (6), we believe that it is worthwhile to explore FMT as a potential new treatment for AS.

## Materials and Methods

### Patient Case

We present a case study of an 18-year-old male patient who was admitted to our hospital in April 2020 after he was diagnosed with AS and diarrhea-predominant irritable bowel syndrome (IBS-D). He was a senior high school student (non-resident) and lived with his parents in Dongguan, China for years. Prior to being admitted, he visited our hospital for almost 6 years with chief complaints of diarrhea and abdominal pain. He had diarrhea 4–5 times a day, with unformed stools that had Bristol stool scores of 6–7. He also experienced frequent abdominal pain that was independent of his eating habits but was aggravated when he was nervous. The patient had a history of AS that was initially diagnosed more than 12 years ago and had been followed-up at the Department of Psychology. He had received non-drug treatment at a local hospital. The patient's AS mainly manifested in the following aspects: had to watch and operate his mobile phone when communicating with people; suddenly felt anxious, lost, or had the impulse to destroy things; and occasionally experienced auditory hallucinations and delusions. Considering that the patient had reached adulthood, we invited a psychiatrist for consultation, who completed the following scales: Hamilton Anxiety Scale (HAMA), Hamilton Depression Scale (HAMD), and Symptom Checklist 90 (SCL-90). Some physical examination were also performed. Blood routine, urine routine, stool routine and liver function were normal. Blood uric acid in renal function was higher (568 μmol/L [normal value: 208–428 μmol/L]), while other renal function indexes (creatinine, glomerular filtration rate and urea) were normal. Electrocardiogram, chest X-ray, abdominal color Doppler ultrasound, brain MRI + DWI and electroencephalogram were normal. Based on a previously described method (7), the patient received FMT once every other day via transendoscopic enteral tubing (TET) over a week, for a total of three rounds. The donor was a 28-year-old male student.

### FMT Procedure

#### TET Tube Insertion

Standard bowel preparation (8) was performed, a colonoscopy was performed to examine the whole colon and distal ileum. A TET tube (FMT Medical Co., Ltd., Nanjing, China) was inserted via the anus as far as the terminus of the ileum, using an endoscope, and the head of the TET tube was fixed with a clamp that was attached to the intestinal wall. An additional two loops of the TET tube were fixed with clamps to the intestinal wall while removing the endoscope. The end of the TET tube was fixed with tape to the sacral skin, as previously described (9, 10).

#### Fecal Microbiota Preparation and Transplantation

One hundred and fifty to two hundred gram fresh feces were collected from a 28-year-old male health donor, dissolved in 1,000 mL physiological saline and was purified by the GenFMTer automatic purification system (FMT Medical Co., Ltd., Nanjing, China), according to the manufacturers protocol. One hundred and fifty milliliter physiological saline containing 50 cm^3^ centrifuged microbiota was infused into the patient via the TET tube. Patient was required to remain in the right lateral position for ≥30 min after transplantation and were allowed to eat 2 h later (to keep the largest contact area between microbiota and intestines). The FMT procedure was repeated every other day for a total of 3 times (9).

### Microbiological and Metabolomics Studies

Using a DNA extraction kit (Tiangen Company, China), total genomic DNA was extracted from approximately 100 mg of stool samples. The concentration and purity of the extracted bacterial DNA were detected using a Qubit 2.0 Fluorometer (Thermo Scientific, USA). Approximately 2 μg of DNA per sample was prepared. Sequence libraries were generated using NEBNext^®^ Ultra™ DNA Library Prep Kit for Illumina (NEB, USA). The libraries were sequenced on the Illumina Hiseq platform at the Novogene Bioinformatics Technology Co., Ltd. (Tianjin, China). Human reads (based on alignment to hg19) or low-quality sequences were discarded, and the high-quality sequences were assembled using SOAPdenovo v2.04 (https://github.com/aquaskyline/SOAPdenovo2) (11). All raw metagenomic data have been submitted to NCBI (accession number: PRJNA701528).

Serum were subjected to metabolomics analysis using liquid chromatography/mass spectrometry (LC/MS) (Gene Denovo Co. Ltd, Guangzhou, China), consistent with metagenomic sequencing. The samples underwent UHPLC-QE Orbitrap/MS analysis (12). The LC/MS analyses were performed according to Li et al. (13). In brief, the UHPLC system (Agilent Technologies), which contained a UPLC HSS T3 column (2.1 mm × 100 mm, 1.7 μm) coupled to a Q Exactive Orbitrap (Thermo Fisher Scientific), was used. Formic acid (0.1%) or ammonium acetate (5 mM) served as solvent A for the positive (ES+) or negative (ES-) ion modes, respectively. Solvent B was acetonitrile. The gradient elution of solvent B was as follows: 1%, 0–1 min; 99%, 8 min; 99%, 10 min; 1%, 10 min; and 1%, 12 min. The Q Exactive Orbitrap mass spectrometer, controlled by Xcalibur v4.0.27, can acquire the full scan survey MS and MS/MS spectra. The spray voltage of ES+ was 3.8 kV, while it was 3.1 kV for ES-. The capillary temperature was 320°C. Approximately 70–1,000 mass-to-charge (m/z) masses were acquired. The resolving power of the full MS and MS/MS was set to 70,000 and 17,500, respectively. The raw data were converted into mzML format using ProteoWizard and preprocessed using R package XCMS v3.2 (14). The processed data included peak intensity, m/z, and retention time. The metabolites were identified via their featured peaks using OSI/SMMS v1.0. Impurity peaks and duplicate identifications were eliminated. For each data set, we removed compounds that were present in <50% of samples within a study. The identification of tentative metabolites were mapped in MS and MS/MS databases using HMDB (https://hmdb.ca) (15).

## Results

### Patient Case

Notably, the patient responded very well to the FMT treatment, with significant improvement in diarrhea and abdominal pain (e.g., defecation times reduced to 1–2 × per day, Bristol stool scores were improved [reduced to 3–4 points], and abdominal pain was relieved). Moreover, the patient's mental state was also significantly improved upon completing the third round of FMT. The effect of the FMT treatment persisted in the 1 month follow-up after discharge (e.g., patient was communicating with people without a mobile phone, the auditory hallucinations and delusions disappeared, and while he occasionally felt lost, he recovered quickly). HAMA, HAMD and SCL-90 showed certain improvement, especially in the following aspects in the 1 week/month follow-up: HAMA (total score: 13-3-2; somatic anxiety: 4-1-0; psychic anxiety: 9-2-2), HAMD (total score: 15-11-9), and SCL-90 (total score: 311-242-232; somatization: 22-18-15; interpersonal sensitivity: 36-30-28; depression: 59-40-43; anxiety: 44-27-24; hostile: 24-18-18; psychotic: 37-24-21). Blood uric acid was lower in the 1 month follow-up (redued to 484 μmol/L). However, 3 months after FMT, the patient's psychological symptoms reappeared. Further inquiry revealed that he broke up with his girlfriend 2 months after the FMT, and his moods fluctuated greatly. Fortunately, his IBS symptoms were still improved compared to before FMT (Table 1).

**Table 1:** Follow up of patient's psychological and digestive symptoms.

		**Pre-FMT**	**Post-FMT (1 wk)**	**Post-FMT (1 mo)**	**Post-FMT (3 mo)**
HAMA	Total score	13	3	2	6
	Somatic anxiety	4	1	0	2
	Psychic anxiety	9	2	2	4
HAMD	Total score	15	11	9	16
SCL-90	Total score	311	242	232	315
	Somatization	22	18	15	26
	Obsessive symptoms	35	27	30	32
	Interpersonal sensitivity	36	30	28	36
	Depression	59	40	43	62
	Anxiety	44	27	24	37
	Hostile	24	18	18	28
	Terrorist	11	12	13	9
	Paranoia	13	18	15	18
	Psychotic	37	24	21	40
	Additional items	30	28	25	27
Digestive symptoms	Frequency of defecation (/d)	4–5	1–2	1–2	3–4
	Bristol Stool Scale	6–7	3–4	3–4	5–6
	Abdominal pain	frequent	seldom	seldom	seldom

*HAMA, hamilton anxiety scale; HAMD, hamilton depression scale; SCL-90, symptom check list-90*.

### Microbiological and Metabolomics Studies

**Figure 1:**
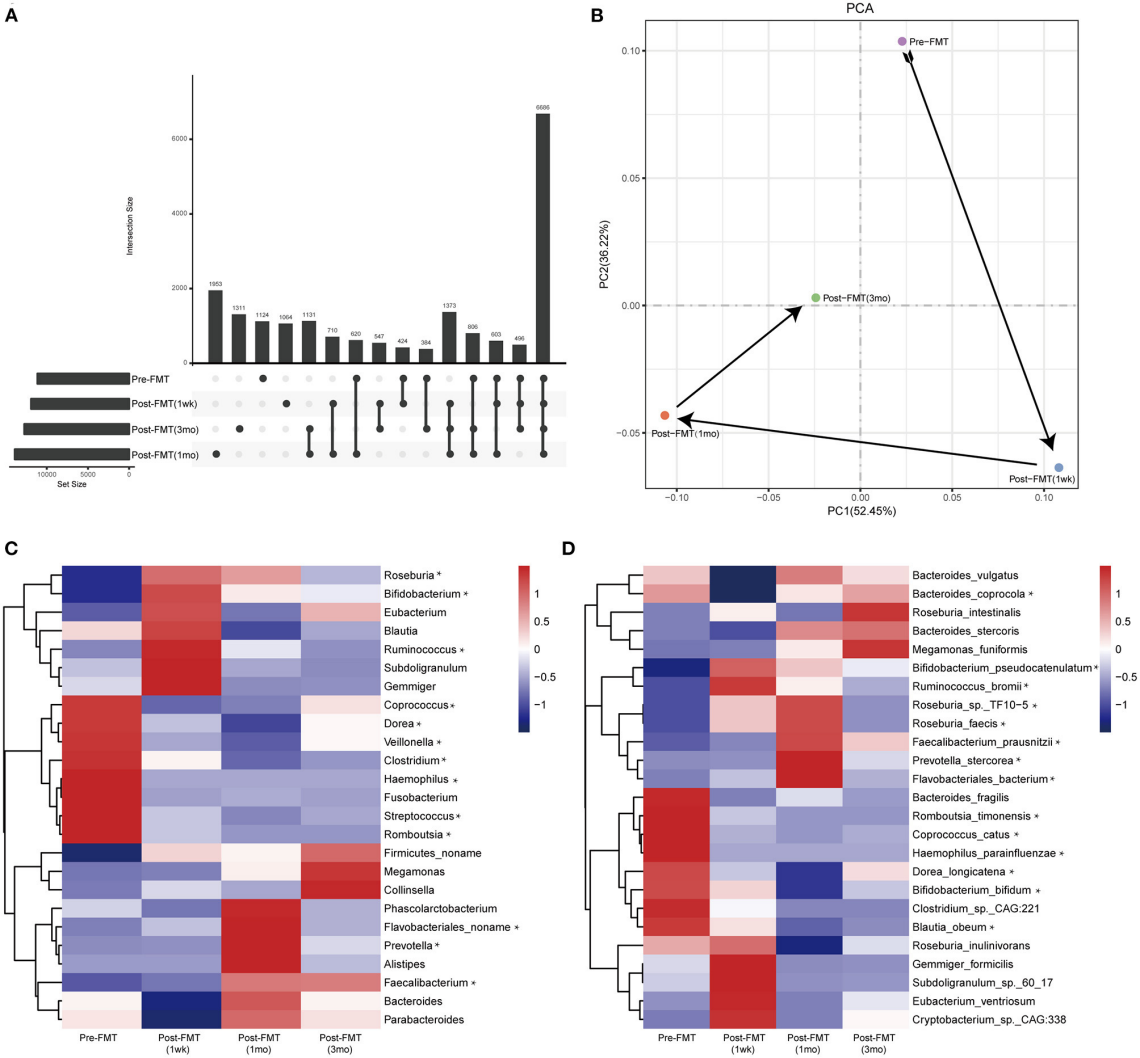
Gut microbiota profiles before and after fecal microbiota transplantation (FMT). **(A)** Venn diagram; **(B)** principal component analysis. Relative abundance of the gut microbiota at the genus **(C)** and species **(D)** levels before and after FMT. *The change trend is the same as the clinical symptoms.

We performed metagenomic sequencing on the fecal samples as well as untargeted metabolomics analysis of the serum samples before and after FMT (i.e., at 1 week, 1 month, and 3 months following FMT). Venn (Figure 1A) and principal component (Figure 1B) analysis revealed that the number of gut microbiota operational taxonomic units changed before and after treatment. Moreover, the microbial structure distance 1 week and 1 month following FMT was different from that before treatment, albeit with a regression 3 months after FMT.

**Figure 2:**
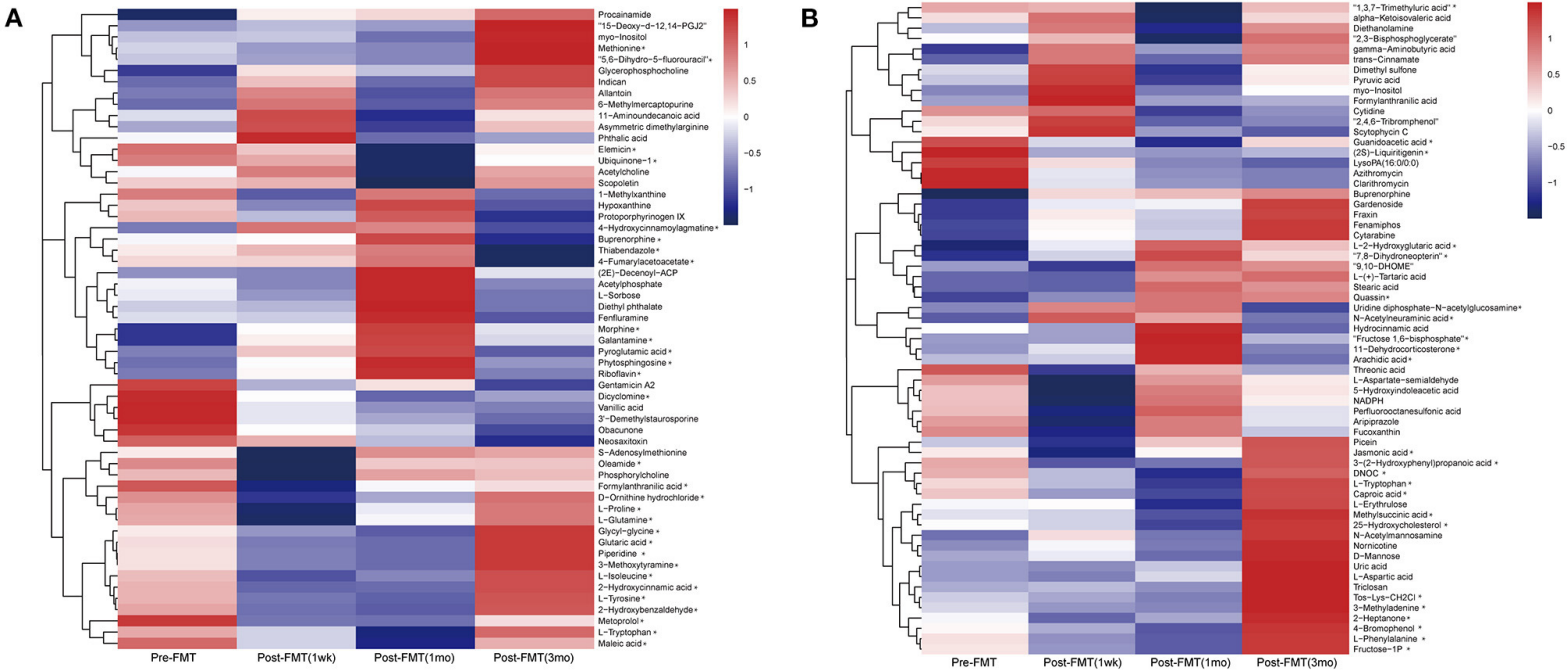
Metabolite profiles before and after fecal microbiota transplantation. The untargeted metabolomics analysis of the serum was conducted under positive **(A)** and negative **(B)** ion state, respectively. *The change trend is the same as the clinical symptoms.

We identified bacteria with similar changes in abundance at the genus (Figure 1C), species (Figure 1D), and metabolite (Figure 2) levels and summarized them based on changes in the patient's clinical symptoms. At the genus level, the relative abundances of *Roseburia, Bifidobacterium, Ruminococcus, Flavobacteriales noname, Prevotella*, and *Faecalibacterium* were increased, while those of *Coprococcus, Dorea, Veillonella, Clostridium, Haemophilus, Streptococcus*, and *Romboutsia* were decreased. At the species level, the relative abundances of *Bifidobacterium pseudocatenulatum, Ruminococcus bromii, Roseburia sp. TF10-5, Roseburia faecis, Faecalibacterium prausnitzii, Prevotella stercorea*, and *Flavobacteriales bacterium* were increased, while those of *Bacteroides coprocola, Romboutsia timonensis, Coprococcus catus, Haemophilus parainfluenzae, Dorea longicatena, Bifidobacterium bifidum*, and *Blautia obeum* were decreased.

The untargeted metabolomics analysis of the serum was conducted under ES+ and ES- ion states. However, due to large changes in serum metabolites and improvement in the patient's symptoms, we selected metabolites that were highly abundant and displayed similar trends. From the content of the first 100 metabolites, we identified those that were differentially expressed. In ES+ mode, 9 metabolites were increased, and 21 metabolites were decreased (Figure 2A), while in ES- mode, 8 and 16 metabolites were increased and decreased, respectively (Figure 2B).

Next, we analyzed the relationship between the 17 increased and 37 decreased metabolites in known signaling pathways using the Kyoto Encyclopedia of Genes and Genomes (KEGG) database (Table 2). We screened for signaling pathways that were associated with at least three metabolites and found that the upregulated metabolites were mainly related to three signaling pathways: ko01100 (Metabolic pathways), ko01110 (Biosynthesis of secondary metabolites), and ko01120 (Microbial metabolism in diverse environments). In contrast, the downregulated metabolites were mainly related to 17 signaling pathways: ko01100 (Metabolic pathways), ko01110 (Biosynthesis of secondary metabolites), ko01120 (Microbial metabolism in diverse environments), ko04974 (Protein digestion and absorption), ko00970 (Aminoacyl-tRNA biosynthesis), ko01230 (Biosynthesis of amino acids), ko05230 (Central carbon metabolism in cancer), ko01130 (Biosynthesis of antibiotics), ko04978 (Mineral absorption), ko01210 (2-Oxocarboxylic acid metabolism), ko00360 (Phenylalanine metabolism), ko00400 (Phenylalanine, tyrosine and tryptophan biosynthesis), ko02010 (ABC transporters), ko00260 (Glycine, serine and threonine metabolism), ko00350 (Tyrosine metabolism), ko00380 (Tryptophan metabolism), and ko00460 (Cyanoamino acid metabolism).

**Table 2:** Metabolites changed after FMT and related signal pathways in this case.

**Increase**		**Decrease**	
**POS & NEG**	**KEGG**	**POS & NEG**	**KEGG**
4-Hydroxycinnamoylagmatine	ko00330	5,6-Dihydro-5-fluorouracil	ko00983
4-Fumarylacetoacetate	ko01100;ko01120;ko00350	Ubiquinone-1	ko01100;ko01110;ko00130;ko00190
Morphine	ko01100;ko01110;ko04080;ko0098	Formylanthranilic acid	ko00380
Galantamine	ko01110	L-Proline	ko01100;ko01110;ko01130;ko01230; ko02010;ko00330;ko04974; ko05230; ko00970;ko04978
Pyroglutamic acid	ko01100;ko00480	L-Glutamine	ko01100;ko01120;ko01230;ko02010; ko00230;ko04974;ko00240;ko05230; ko00970;ko00630;ko00250;ko04978; ko00220;ko04727;ko04964;ko00471; ko00910;ko04724
Phytosphingosine	ko01100;ko00600	Glutaric acid	ko01120;ko00310;ko00071
Riboflavin	ko01100;ko01110;ko04977;ko00740	Piperidine	ko04974
L-2-Hydroxyglutaric acid	ko01120	3-Methoxytyramine	ko01100;ko00350;ko04728
7,8-Dihydroneopterin	ko01100;ko00790	L-Isoleucine	ko01100;ko01110;ko01130;ko01230; ko02010;ko01210;ko04974;ko05230; ko00970;ko04978;ko00460;ko00290; ko00280
Uridine diphosphate-N-acetylglucosamine	ko01100;ko01130;ko00520;ko00524; ko04931	2-Hydroxycinnamic acid	ko01110;ko01120;ko00360
N-Acetylneuraminic acid	ko01100;ko00520	L-Tyrosine	ko01100;ko01110;ko01130; ko01230;ko00350;ko01210;ko04974; ko05230;ko00360;ko00970;ko00400; ko00130;ko00460;ko00261;ko00730; ko05034;ko04728;ko05012;ko05030; ko05031;ko04917;ko04916
Fructose 1,6-bisphosphate	ko0110;ko01110;ko01120;ko01130; ko01200;ko05230; ko00400;ko0492;ko04152	2-Hydroxybenzaldehyde	ko01100;ko01120;ko0122
11-Dehydrocorticosterone	ko00140	L-Tryptophan	ko01100;ko01110;ko01130;ko01230; ko01210;ko04974;ko05230;ko00380; ko00260;ko00970;ko00400;ko04978; ko04726;ko05143
Arachidic acid	ko01040	Maleic acid	ko01120;ko00350;ko00760;ko00650
		1,3,7-Trimethyluric acid	ko01120;ko00232
		Guanidoacetic acid	ko01100;ko00330;ko00260
		(2S)-Liquiritigenin	ko01110
		Jasmonic acid	ko01100;ko01110;ko00592
		3-(2-Hydroxyphenyl)propanoic acid	ko01120;ko00360
		L-Tryptophan	ko01100;ko01110;ko01130;ko01230; ko01210;ko04974;ko0523;ko00380; ko00260;ko00970;ko00400;ko04978; ko04726;ko05143
		25-Hydroxycholesterol	ko00120
		4-Bromophenol	ko00980
		L-Phenylalanine	ko01100;ko01110;ko01130;ko01230; ko02010;ko01210;ko04974;ko05230; ko00360;ko00970;ko00400;ko04978; ko00460

## Discussion and Conclusion

To our knowledge, this is the first report in which AS was successfully ameliorated using FMT. More specifically, FMT markedly improved the symptoms of AS along with diarrhea and abdominal pain. As we were intrigued by the findings, we further explored how FMT exerted its therapeutic role in AS. Our findings suggest that FMT may alleviate AS via restoring the gut microbiota, mainly by enhancing the abundance of bacteria associated with short chain fatty acids (SCFAs), such as *Roseburia, Bifidobacterium, Ruminococcus, Prevotella*, and *Faecalibacterium* (16, 17).

We also found changes in serum metabolites following FMT, which may provide some references for future research. The 17 increased and 37 decreased serum metabolites may be related to the patient's symptoms. Enrichment analysis of these 54 metabolites using the KEGG database indicated that they were related to the metabolism of the organism/bacteria. This finding suggests that although FMT directly affects the microbial structure, the metabolites of intestinal microbiota may influence remote organs, such as the nervous system, via the blood circulation, where they then mitigate the mental and psychological symptoms. Accordingly, in future studies on FMT treatments of AS, the focus should include changes in these metabolites and their signaling pathways. Of course, considering that changes in intestinal microbiota seems to be related to SCFAs, the assaying of SCFAs in blood and feces may be more informative.

However, several limitations of the present study should be noted. First, although we carefully analyzed the possible mechanism of FMT in the treatment of AS by using metagenomics and non-target metabonomics, as a case study, the strength of our data is still limited, and more clinical studies and animal experiments are needed for additional verification. Second, although we found that the changes of serum metabolites were related to the intestinal microbiota (especially SCFAs producing bacteria), the specific pathways through which these identified serum metabolites as well as SCFAs affect brain function of AS still need to be further studied. Third, some non-typical core symptoms of AS like occasionally experienced auditory hallucinations and delusions were found in this case, diagnostic process for other mental disorders should been performed and possible comorbid disordered should be excluded. Nevertheless, we hope that the metagenomic data of the case presented here may be a reference for psychiatrists and physicians. The therapeutic effect of FMT in AS makes it a novel option for physicians in creating treatment plans for AS patients.

## Data Availability Statement

The datasets presented in this study can be found in online repositories. The names of the repository/repositories and accession number(s) can be found at: https://www.ncbi.nlm.nih.gov/, PRJNA701528.

## Ethics Statement

Committee of Guangzhou First People's Hospital approved the study. All methods were performed in accordance with the provisions of the Declaration of Helsinki of 1975. Before screening, we gained written informed consents from the participant. Written informed consent was obtained from the patient for publication of the case and any accompanying data.

## Author Contributions

H-LH involved in design of the study, recruitment of the patient, and drafting of the article. H-MX involved in statistical analysis and interpretation of the data and drafting of the article. Y-DL and D-WS performed the sample collection and DNA extraction. H-TC and Y-QN prepared fecal samples into filtrate for administration during the FMT procedure and revision of the article. Y-QL and Y-JZ designed and organized the study, interpretation of the data, and revision of the article. All authors read and approved the final manuscript.

## Funding

This work was supported by the grants from the National Natural Science Foundation of China (81970507), Guangzhou Planned Project of Science and Technology (201904010132 and 202002030288), and Innovative Clinical Technique of Guangzhou (2019GX05).

## Conflict of Interest

The authors declare that the research was conducted in the absence of any commercial or financial relationships that could be construed as a potential conflict of interest.

## Publisher's Note

All claims expressed in this article are solely those of the authors and do not necessarily represent those of their affiliated organizations, or those of the publisher, the editors and the reviewers. Any product that may be evaluated in this article, or claim that may be made by its manufacturer, is not guaranteed or endorsed by the publisher.
